# Single Nucleotide Polymorphisms, Gene Expression and Economic Evaluation of Parameters Associated with Mastitis Susceptibility in European Cattle Breeds

**DOI:** 10.3390/vetsci9060294

**Published:** 2022-06-14

**Authors:** Ahmed I. Ateya, Samer S. Ibrahim, Mona M. Al-Sharif

**Affiliations:** 1 Department of Husbandry & Development of Animal Wealth, Faculty of Veterinary Medicine, Mansoura University, Mansoura 35516, Egypt; samersamir@mans.edu.eg; 2 Department of Biology, College of Science, University of Jeddah, Jeddah 21589, Saudi Arabia; mmalshreef@uj.edu.sa

**Keywords:** Holstein, Brown Swiss, single nucleotide polymorphisms, gene expression, economic evaluation

## Abstract

The objective of this study was to explore single nucleotide polymorphisms (SNPs), gene expression and economic evaluation of parameters associated with mastitis susceptibility in Holstein and Brown Swiss dairy cows. Two hundred and forty Holstein and Brown Swiss dairy cows (120 cows of each breed) were used in this study. The investigated dairy cows in each breed were allocated into two equal-sized groups (60 cows each); mastitis tolerant and affected groups. PCR-DNA sequencing of *SELL*, *ABCG2*, *SLC11A1*, *FEZL*, *SOD1*, *CAT*, *GPX1*, and *AhpC/TSA* revealed nucleotide sequence variations in the form of SNPs associated with mastitis tolerance/susceptibility in investigated Holstein and Brown Swiss dairy cows. Levels of *SELL*, *SLC11A1* and *FEZL* gene expression were significantly up-regulated in mastitic Holstein and Brown Swiss dairy cows than in tolerant ones. Meanwhile, *ABCG2*, *SOD1*, *CAT*, *GPX1*, and *AhpC/TSA* genes were significantly downregulated. Regarding the economic parameters, significant differences were recorded for net returns and a reduction in the percentage of net profit, as the higher values of net returns were recorded for tolerant dairy cows than mastitic ones in both breeds; moreover, the net profit was reduced by 39% and 27% in mastitic Holstein and Brown Swiss dairy cows, respectively, when compared to tolerant ones. The results herein confirmed the potential significance of investigated genes as candidates for mastitis tolerance/susceptibility in Holstein and Brown Swiss dairy cows. Mastitis also has detrimental impacts on economic efficiency in dairy farms.

## 1. Introduction

Mastitis is an inflammatory and important economic disease of the livestock that occurs in response to a bacterial, chemical, thermal or mechanical injury [1]. Following infection of the mammary gland, macrophages and epithelial cells release cytokines that cause the migration of neutrophils, monocytes and other leukocytes from the blood to the site of infection in the mammary tissue. Mastitis is also considered one of the most costly production-related diseases in the dairy industry [2]. The cost associated with mastitis in Europe, according to current estimates, is EUR 1.55 billion per year (European Union http://www.sabre-eu.eu/ (accessed on 1 January 2020)). Economic losses due to mastitis may reach up to USD 35 million, per year, worldwide [3,4]. The frequency and cost of mastitis, and rising public concerns for animal welfare, have made mastitis one of the most important diseases for the dairy sector [5].

Mastitis is a costly disease due to the increasing expenditure to reduce its level and reduction of output. Mastitis is responsible for 38% of total direct losses, and remains the most common disease of economic importance in the dairy industry. Subclinical mastitis has 70% of total financial losses that are associated with decreased milk production, deterioration of milk quality, costs of health management, milk disposal, advanced culling, and the addition of labour requirements [6,7]. The economic loss estimates of clinical mastitis differ largely between farms [8]; mastitis causes also serious economic losses in the dairy sector from the most to the least organised herds [9]. Mastitis is a multifactorial disease, closely related to the environment in which the cows are kept, and its severity can be mild, moderate and severe or permanent [10]. For all decision-makers, the economic benefits and incentives induce the perfection of mastitis management. [11]. There are certain other limitations as the evolvement of antibiotic-resistant mutant bacteria by genetic shift and genetic drift, and therefore vaccinations are not 100% effective; studies have been attempted with bovine mastitis prevention by inoculation of lactic acid bacteria at the dry-off period [12].

The immunity is divided into two, namely the innate and adaptive immunity, in mammals. Innate immunity is present both in vertebrates and invertebrates, whereas adaptive immunity is only present in vertebrates [13]. Host genetic resistance is mainly sustained by innate immunity, providing protection against pathogens without being vaccinated or exposed to diseases [14]. Despite the worldwide efforts to improve herd management practices, mastitis control in the dairy industry remains inadequate. Thus, there is a high demand for measures which would allow us to reduce the incidence of this disease. According to Heringstad et al. (2003), genetic improvement of mastitis resistance can be achieved by traditional breeding [15]; however, it is also known that this trait is lowly heritable and unfavourably correlated with milk production traits [16,17]. Resistance to mastitis is a complex function involving various pathways contributed by numerous candidate genes [18]. Recent developments in genome sequencing technologies applied to livestock have facilitated the identification of copy number variations (CNVs) and millions of SNPs in a relatively cost-efficient manner. This has enabled researchers to describe the genomic landscape of livestock species [19,20,21], and to combine these whole-genome sequence data with phenotypic information for genomic prediction [22] or in genome-wide association studies to identify variations associated with various traits [23,24].

Although there is little information on *SELL*, *ABCG2*, *SLC11A1*, and *FEZL* gene polymorphisms and their association with mastitis susceptibility in dairy cattle, previous studies reported this association in one breed using RFLP and SSCP genetic markers with opposing results [25,26,27,28,29]. Based on the current knowledge, there were no previous studies revealing the association of SNPs and expression profile of immune and antioxidant markers in two breeds of dairy cows. Consequently, the aim of the current work was to revealing the association of SNPs and expression profile of *SELL*, *ABCG2*, *SLC11A1*, *FEZL***,** *SOD1***,** *CAT***,** *GPX1*, and *AhpC/TSA* genes with mastitis tolerance/susceptibility in Holstein and Brown Swiss dairy cows. Another aim was to evaluate some economic parameters associated with mastitis susceptibility in these two breeds.

## 2. Material and Methods

### 2.1. Animals and Experimental Samples

In total, 240 Holstein and Brown Swiss dairy cows (120 cows of each breed) were used in this study. Animals belonged to the same private farm located at Ismailia desert road, Ismailia Governorate, Egypt and shared the same environment. The experiment was carried out between December 2021 and February 2022. Animals were in the third lactation season and were raised in a commercial dairy herd of approximately 450 animals. Cows were 3 years of age on average and 450 kg of average body weight. Animals were housed in a cubicle (free-stall/feedlot) barn with straw-bedded stalls, and a slatted floor that was scraped regularly; they were fed a total mixed ration (TMR), milked twice/day and artificially inseminated. The investigated dairy cows were subjected to thorough clinical examination according to the standard protocols. The investigated dairy cows in each breed were allocated into two equal-sized groups (60 cows each) according to their health status and incidence of mastitis. The first group included clinically healthy cows and was assigned as mastitis tolerant group (have a previous history of mastitis resistance in the previous lactations, i.e., mastitis never observed in the previous lactations). The second group comprised cows demonstrating mastitis and was assigned as mastitis affected group (high body temperature, low appetite, swollen and tender udder, reddish and yellowish milk colour and bad odour, clotted milk, teat cracks). California mastitis test was also used for screening for the mastitis incidence in the investigated cows). Five millilitres of blood was collected from each cow in all groups via jugular vein puncture. The samples were collected into a vacutainer tube containing EDTA as an anticoagulant to yield whole blood for DNA and RNA extraction. Blood samples were kept frozen at −20 °C until subsequent DNA extraction. To avoid hydrolysis of RNA, freshly collected blood samples were sent without delay for RNA extraction. All procedures were performed in accordance with the guidelines of Mansoura University and were approved by the Ethical Committees. Research Ethics Committee, Faculty of Veterinary Medicine, Mansoura University approved the protocol of the study (code R/124).

### 2.2. DNA Extraction and Polymerase Chain Reaction (PCR)

Genomic DNA was extracted from whole blood was done using a Gene JET whole blood genomic DNA extraction kit following the manufacturer procedure (Thermo Scientific, Lithuania). The quality, purity, and concentration of DNA were assessed using a Nanodrop before further analysis. PCR was carried out for amplification of fragments of *SELL*, *ABCG2*, *SLC11A1*, *FEZL***,** *SOD1***,** *CAT***,** *GPX1*, and *AhpC/TSA* genes. The primer sequences were designed according to the PubMed published sequence of *Bos taurus*. The primers used in the amplification are shown in Table 1. The polymerase chain reaction mixture was done in a final volume of 100 μL in a thermal cycler. Each reaction volume contained 5 μL DNA, 43 μL H_2_O (d.d water), 50 μL PCR master mix (Jena Bioscience, Germany), and 1 μL of each primer. The reaction mixture was subjected to an initial denaturation temperature of 95 °C for 4 min. The cycling proceeded for 35 cycles of 95 °C for 1 min for denaturation, one minute for annealing temperatures (as shown in Table 1), extension at 72 °C for 1 min and a final extension at 72 °C for 10 min. Samples were held at 4 °C. Representative PCR results were detected by agarose gel electrophoresis, and then fragment patterns were visualized under UV using a gel documentation system.

### 2.3. DNA Sequencing and Polymorphism Detection

Before DNA sequencing, removing primer dimmers, nonspecific bands and other impurities was done. As described by Boom et al. [30] purification of PCR products with the expected size (target bands) was carried out using a PCR purification kit following the manufacturer procedures (Jena Bioscience # pp-201×s/Germany, Jena, Germany). Quantification of PCR product was carried out using Nanodrop (Uv-Vis spectrophotometer Q5000/USA) in order to yield high products and to ensure enough concentrations and purity of the PCR products [31]. To detect SNPs in tolerant and mastitis-affected dairy cows, PCR products with target band were sent for DNA sequencing in forward and reverse directions. These products were sequenced with an ABI 3730XL DNA sequencer (Applied Biosystems, Waltham, MA, USA) according to the enzymatic chain terminator technique developed by Sanger et al. [32]. 

DNA sequencing data were analyzed with Chromas 1.45 and BLAST 2.0 software [33]. Differences were classified as SNPs between PCR products of studied genes and reference sequences available in GenBank. On the basis of an alignment of sequences, variation of amino acid sequence of the investigated genes between enrolled dairy cows was identified using the MEGA4 software [34].

### 2.4. Total RNA Extraction, Reverse Transcription and Quantitative Real-Time PCR

Total RNA was extracted from the blood of investigated dairy cows using Trizol reagent following the manufacturer’s instructions (RNeasy Mini Ki, Catalogue No. 74104). The amount of extracted RNA was quantified and qualified using NanoDrop^®^ ND-1000 Spectrophotometer. The cDNA of each sample was synthesized following the manufacture protocol (Thermo Fisher, Catalog No, EP0441, Waltham, MA, USA). Assessment of the expression pattern of *SELL*, *ABCG2*, *SLC11A1*, *FEZL***,** *SOD1***,** *CAT***,** *GPX1*, and *AhpC/TSA* genes was carried out using quantitative RT-PCR using SYBR Green PCR Master Mix (2x SensiFastTM SYBR, Bioline, CAT No: Bio-98002, Toronto, ON, Canada). Relative quantification of mRNA level was performed by real-time PCR using SYBR Green PCR Master Mix (Quantitect SYBR green PCR kit, Catalog No, 204141). Primer sequences were designed according to the PubMed published sequence of *Bos taurus*, as shown in Table 2. The housekeeping gene *ß. actin* was used as a constitutive control for the normalisation. The reaction mixture was carried out in a total volume of 25 µL consisting of total RNA 3 µL, 4 µL 5 × Trans Amp buffer, 0.25 µL reverse transcriptase, 0.5 µL of each primer, 12.5 µL 2 × Quantitect SYBR green PCR master mix and 8.25 µL RNase free water. The final reaction mixture was placed in a thermal cycler and the following program was carried out: reverse transcription at 50 °C for 30 min, primary denaturation at 94 °C for 8 min followed by 40 cycles of 94 °C for 15 s, annealing temperatures as shown in Table 2, and 72 °C for 30 s. At the end of the amplification phase, a melting curve analysis was performed to confirm the specificity of the PCR product. The relative expression of each gene per sample in comparison with *ß. actin* gene was carried out and calculated according to the 2^−ΔΔCt^ method [35,36].

### 2.5. Economic Parameters

The economic data were obtained from the accurate farm records and the structured questionnaire method

#### 2.5.1. Total Variable Costs (TVC)

Total variable costs (TVC) included labor, feed, veterinary management and uncertainly costs that include the dead animal value and costs related to production [37]. Service costs were calculated as: services number till conception × cost of one service.

#### 2.5.2. Total Fixed Costs (TFC)

Total Fixed costs (TFC) included land, building and equipment depreciation. The buildings depreciation rate was calculated on the basis of 25 years, whereas the equipmentdepreciation was calculated on the basis of 5 years [38]. The rent value was used directly during the calculation in cases where the farms are not owned [39].

Depreciation rate = value of asset / age of asset (year).

#### 2.5.3. Total Costs (TC)

The total costs (TC) included the sum of total variable costs and total fixed costs [40].

TC = TVC + TFC.

#### 2.5.4. Total Return

The total return was calculated by the equation described by Fidan, 2010 [41]. All prices were also determined according to the market price during the study period.

Total return (TR) = total milk sale (kg) + return of new calves sale + return of fecal matter sale (m^3^).

#### 2.5.5. Net Income (Net Return)

The net income was calculated by the following equation as described by Atallah, 2004 [42].

Net income = total return−total costs.

#### 2.5.6. Reduction Percentage in Net Profit

The reduction percentage of net profit was calculated through the difference between the net return in healthy animals and that in mastitic animals for each breed. Then, the latter difference is compared to the net return of healthy dairy cows to know the percentage and to evaluate the impact of mastitis on the profitability of dairy cows [43].

### 2.6. Statistical Analysis

Ho: Single nucleotide polymorphisms, gene expression and economic evaluation approaches could not explore mastitis tolerance/susceptibility in Holstein and Brown Swiss dairy cows.

HA: Single nucleotide polymorphisms, gene expression and economic evaluation approaches could explore mastitis tolerance/susceptibility in Holstein and Brown Swiss dairy cows.

Statistical parameters were expressed as mean ± standard deviation (SD). Data were statistically analysed using SPSS program version 23 (One-way ANOVA test for comparing between the studied groups’ mean and multiple comparison Tukey‘s HSD test for estimation of post-hoc differences between means). A difference was considerably significant at P< 0.05. Chi-square analysis was carried out to assess the significant distribution in identified SNPs of genes between the total two hundred and forty (120 of each breed) mastitis tolerant and affected dairy cows. A discriminant analysis model was carried out to check the significance of different determinants to classify tolerant and affected dairy cows as a dependent variable using the gene expression profile of investigated genes as an independent variable. To discriminate between mastitic and tolerant cows based on expression profile of investigated genes, a univariate general linear model (GLM) was used to test the interaction effect of gene type and mastitis tolerance/susceptibility on gene expression results parameter, where data represented this as a mean ± SE.

## 3. Results

### 3.1. PCR-DNA Sequencing of Investigated Genes

PCR-DNA sequencing of *SELL* (809-bp), *ABCG2* (756-bp), *SLC11A1* (450-bp), *FEZL* (813-bp), *SOD1* (334-bp), *CAT* (268-bp), *GPX1* (534-bp), and *AhpC/TSA* (480-bp) revealed nucleotide sequence variations in the form of SNPs associated with mastitis tolerance/susceptibility in investigated Holstein and Brown Swiss dairy cows. Nucleotide sequence variation of the investigated genes between tolerant and affected dairy cows and reference sequences available in GenBank confirmed all identified SNPs (Figures S1–S8). Chi-square analysis revealed a significant distribution in the identified SNPs; where a significant difference was detected in frequencies of the investigated genes between the tolerant and affected Holstein and Brown Swiss dairy cows (Table 3).

### 3.2. Gene Expression Pattern of Immune and Antioxidant Markers

**Figure 1:**
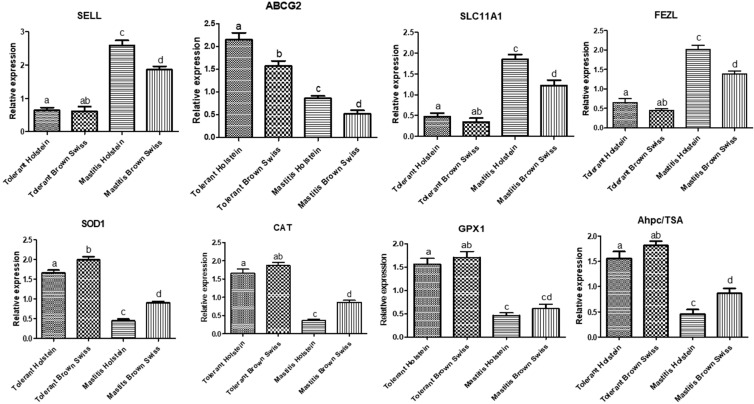
Relative expression patterns of *SELL*, *ABCG2*, *SLC11A1*, *FEZL*, *SOD1*, *CAT*, *GPX1*, and *AhpC/TSA* genes in the tolerant and mastitis-affected Holstein and Brown Swiss dairy cows. a, b, c, d, ab, cd means small alphabetic letters show significance when *p* < 0.05.

The gene expression profile of immune and antioxidant markers is depicted in Figure 1. Levels of *SELL*, *SLC11A1* and *FEZL* gene expression were significantly up-regulated in mastitic Holstein and Brown Swiss dairy cows in comparison to tolerant ones; meanwhile, *ABCG2***,** *SOD1***,** *CAT***,** *GPX1*, and *AhpC/TSA* genes were significantly down-regulated.

There was a significant interaction between the type of gene and mastitis tolerance/susceptibility in each breed on mRNA levels of investigated markers. Among all genes evaluated in the mastitis-affected dairy cows, the highest possible level of mRNA was identified for *SELL* in Holstein (2.58 ± 0.15) and Brown Swiss (1.85 ± 0.11) dairy cows; whereas, the lowest level was for *CAT* (0.36 ± 0.03) and *ABCG2* (0.52 ± 0.07) in Holstein and Brown Swiss, respectively. In the same respect, among all genes evaluated in the tolerant dairy cows, the highest possible level of mRNA was identified for *ABCG2* (2.15 ± 0.15) and *SOD1* (1.98 ± 0.08) in Holstein and Brown Swiss dairy cows, respectively; whereas, the lowest level was for *SCL11A1* in Holstein (0.47 ± 0.08) and Brown Swiss (0.34 ± 0.09).

### 3.3. Economic Evaluation of Parameters Associated with Mastitis Susceptibility in Holstein and Brown Swiss Breeds

The results depicted in Table 4 cleared that service, treatment, total veterinary management and the total costs differed significantly among tolerant and mastitic Holstein and Brown Swiss dairy cows (*p* < 0.05). The higher total costs were recorded in mastitic Holstein dairy cows than both tolerant and mastitic Brown Swiss ones. The total returns decreased in the mastitic dairy cows compared to the healthy ones in both breeds.

Significant differences were recorded for net returns and reduction in the percentage of net profit, as the higher values of net returns were recorded for tolerant dairy cows than mastitic ones in both breeds; moreover, the net profit was reduced by 39% in mastitic Holstein cow when compared to tolerant ones; in contrast, the net profit was reduced by 27% in mastitic Brown Swiss dairy cows in comparison to tolerant ones.

## 4. Discussion

The major concern of breeding is getting more robust cows [44]. Until recently, less attention has been paid to health traits, including immune response as a result of livestock species, rather they have been mainly selected for production traits [15]. Genetic improvement of mastitis resistance can be achieved by traditional breeding by the selection of favourable animals on the basis of phenotype; however, it is also known that this trait is lowly heritable and unfavourably correlated with milk production traits [16,17]. The candidate gene approach focuses more on identifying genes that are connected to mastitis through activities such as recognition of pathogens, leukocyte recruitment, migration, pathogen elimination and resolution [45,46].

In this context, PCR-DNA sequencing for fragments of *SELL* (809-bp), *ABCG2* (756-bp), *SLC11A1* (450-bp), *FEZL* (813-bp), *SOD1* (334-bp), *CAT* (268-bp), *GPX1* (534-bp), and *AhpC/TSA* (480-bp) genes revealed nucleotide sequence variations in the form of SNPs between mastitis tolerant and affected Holstein and Brown Swiss dairy cows. Chi-square analysis revealed a significant distribution in the identified SNPs. As far as we are concerned, this is the first study revealing SNPs in *SELL*, *ABCG2*, *SLC11A1*, *FEZL*, *SOD1*, *CAT*, *GPX1*, and *AhpC/TSA* genes as candidates for mastitis tolerance/susceptibility in Holstein and Brown Swiss dairy cows. Interestingly, our results indicated that the polymorphisms identified are reported here for the first time in investigated genes when compared with matched GenBank reference sequence. Although, there is little information on *SELL*, *ABCG2*, *SLC11A1*, and *FEZL* genes polymorphisms and their association with mastitis susceptibility in dairy cattle, previous studies reported these association in one breed using RFLP and SSCP genetic markers with opposing results [25,26,27,28,29]. Unlike previous studies, this context explored polymorphisms via SNP genetic marker to compare mastitis incidence in two breeds (Holstein and Brown Swiss) of dairy cattle. SNP genetic marker revolutionizes previous achievements in conservation decisions, biodiversity assessment and genetic characterization of breeds [47]. SNPs analysis could also explain the history of European cattle more accurately than other markers [48,49,50,51]. Particular importance is also attributed to SNPs in the search for linkages between a marker with a specific location in the genome and an unknown gene locus. The search for such associations is important because they allow a phenotypic effect to be assessed by identifying its genetic basis [49,51].

Dusza et al. [27] studied *SELL* gene polymorphism and its association with clinical mastitis and milk production in Polish Holstein-Friesian cattle. Strong associations were observed between *SELL* gene polymorphism and milk production traits (milk yield, milk fat percentage, and milk protein percentage); however, the polymorphism in the analysed gene had no influence on the resistance or susceptibility of cows to clinical mastitis. Chen et al. [52] reported also polymorphisms in the *SELL* gene cluster were associated with fertility and survival time in Holstein Friesian cows. Yue et al. [25] reported an association between ABCG2 gene polymorphisms and somatic cell scores (SCS) in Holstein cattle; however, contrasting results were reported by Asadollahpour et al. [26] who elaborated that genetic polymorphisms in the ABCG2 gene had no effect on SCS. Komisarek et al. [53] reported a significant effect of *ABCG2* gene polymorphisms on estimated breeding values for milk fat production traits, calving-to-first insemination interval, and non-return rate in Polish Holstein-Friesian cattle. Genetic effects of *ABCG2* polymorphism on milk production traits were also reported in the Holstein cattle [54,55]. Zhang et al. [28] elicited that *SLC11A1* gene polymorphisms are not associated with somatic cell score and milk yield in Chinese Holstein. *SLC11A1* gene polymorphisms were also associated with tolerance /susceptibility to bovine tuberculosis [56,57]. Ghada et al. [29] reported an association between *FEZL* gene polymorphisms and mastitis susceptibility in Holstein cattle. Somasundaram et al. [58] also indicated that polymorphism of the *FEZL* gene was associated with mastitis resistance in Indian cattle. Darwish et al. [59] indicated that SNPs in the *SOD* gene were associated with postpartum disorders susceptibility in Barki sheep.

In the present study, a real-time PCR was carried out to quantify the mRNA level of *SELL*, *ABCG2*, *SLC11A1*, *FEZL*, *SOD1*, *CAT*, *GPX1*, and *AhpC/TSA* genes in tolerant and susceptible Holstein and Brown Swiss dairy cows to mastitis. Our findings revealed that the Gene expression profile of *SELL*, *SLC11A1* and *FEZL* genes expression were significantly up-regulated in mastitic Holstein and Brown Swiss dairy cows than tolerant ones; meanwhile, *ABCG2*, *SOD1*, *CAT*, *GPX1*, and *AhpC/TSA* genes were significantly downregulated. Our study is the first to detect immune and antioxidant mRNA levels in tolerant and susceptible Holstein and Brown Swiss dairy cows to mastitis using real-time PCR approach. Previous studies explored gene polymorphism using genetic markers such as RFLP and SSCP [25,26,27,28,29]; however, our study was designed to overcome the limitations of previous work by investigating polymorphism in studied genes using SNP genetic markers and gene expression. Consequently, *SELL*, *ABCG2*, *SLC11A1*, *FEZL*, *SOD1*, *CAT*, *GPX1*, and *AhpC/TSA* regulation mechanisms are well understood in the mastitis tolerant and affected Holstein and Brown Swiss dairy cows. To the best of our knowledge, this study is the first that declared the gene expression profile of immune and antioxidant markers associated with mastitis tolerance/susceptibility in dairy cattle. In terms of the gene expression profile of immune and antioxidant markers in ruminants, Darwish et al. [59] elicited that mRNA levels of *SOD* and *CAT* genes were significantly downregulated in ewes affected with postpartum disorders than in tolerant ones. Asadpour et al. [60] studied a differential expression of the antioxidant genes during clinical mastitis of cows caused by Staphylococcus aureus and Escherichia coli, where the expression profile of *SOD* in mastitis milk induced by S. aureus was significantly up-regulated compared with E. coli. In addition, the mRNA levels of GPx in mastitis milk due to E. coli were significantly over expressed compared to S. aureus. Ateya et al. [61] elaborated that the profound alteration in the gene expression profile of antioxidant genes could be a biomarker that helps follow-up health during the peri-parturient period in dromedary camels.

L-selectin is one of the most important adhesion molecules expressed on polymorphnuclear cells; it is coded by the *SELL* gene, which is responsible for neutrophil attachment to endothelium [62]. L-selectin mediates the migration of activated circulating PMN across the blood–milk barrier in the process of diapedesis through the endothelium of the mammary gland [63]. ATP binding cassette sub family G member 2 (ABCG2) gene encodes a transporter protein that facilitates the transport of medicines through the cell membrane by binding ATP; it has a crucial role in the protection of various cells and tissues against xenotoxins and/or endotoxins [64]. The expression of this gene in the mammary significantly increases during lactation compared to the dry period [65] which could decipher its up-regulation in tolerant Holstein and Brown Swiss dairy cows rather than mastitic ones. Solute Carrier Family 11 Member 1 (SLC11A1) is a trans-membrane protein and was reported to be one of the best-known potential candidate genes that promote innate immunity against different intracellular pathogens [66]. Forebrain embryonic zinc finger-like gene (*FEZL*) was identified as a QTL influencing mastitis resistance [67]; it also has an immune function, as it plays an important antimicrobial role by controlling the neutrophilic migration to the site of mammary gland infection [68]. Semaphorine 5A (*SEMA5A*) is one of the target genes of FEZL and is represented as an extensive family of widely expressed secreted and membrane-associated proteins [69]. When cows are infected with mastitis, FEZL, as a transcription factor, is able to induce tumour necrotic factor-α (TNF-α) and interleukin-8 (IL-8) through enhancing SEMA5A [70].

The field of oxidative stress in ruminant medicine is still in the early stages of development. The protective mechanisms of antioxidants are to scavenge or detoxify ROS, block their production, or sequester transition metals that are the source of free radicals [71]. These mechanisms include enzymatic and non-enzymatic antioxidant defenses produced in the body, namely, endogenous as superoxide dismutase (SOD), catalase (CAT) and glutathione peroxidase (GPx) [72] and others supplied with the diet, namely, exogenous as polyphenols [73]. Alkyl hydroperoxide reductase/Thiol-specific antioxidant (AhpC/TSA) plays a role in cell protection against oxidative stress by detoxifying peroxides and sulphur-containing radicals, and acts as a sensor of hydrogen peroxide-mediated signalling events [74]. Despite oxidative stress has been associated with numerous conditions, there is a great deal to be discovered about its role in ruminant health and production. Measuring of enzymes and vitamins antioxidants in biological samples is considered also one of the popular methods for the detection of oxidative stress [75]. The marked alteration of the expression pattern of immune (*SELL*, *ABCG2*, *SLC11A1*, and *FEZL*) and antioxidant (*SOD1*, *CAT*, *GPX1*, and *AhpC/TSA*) markers in dairy cows with mastitis may be attributed to severe inflammation that damages the affected tissue and cytotoxic radicals and pro-inflammatory cytokines that are released by the phagocytic cells [76,77]; moreover, the extra quantity of ROS in the absence of an optimal total antioxidant leads to the predominance of ROS, and compromises the immune system [78].

Regarding the economic evaluation of parameters associated with mastitis susceptibility. The higher total costs were recorded in mastitic Holstein dairy cows than both tolerant and mastitic Brown Swiss ones. The total returns decreased in the mastitic dairy cows compared to the healthy ones in both breeds. Significant differences were recorded for net returns and reduction in net profit percentage, as the higher values of net returns were recorded for tolerant dairy cows than mastitic ones in both breeds; moreover, the net profit was reduced by 39% and 27% in mastitic Holstein and Brown Swiss dairy cows, respectively, when compared to tolerant ones. These results may be attributed to the high costs of drugs, disinfectants and veterinary supervision used in the treatment course of diseased animals [7]; moreover, the lower total costs in mastitic Brown Swiss cows compared with other cows is owing to their resistance being slightly higher than Holstein, and thus the rapid recovery of diseased animals [79]. Our results are in harmony with those of Eltarabany and Ali [11] who concluded that the total variable costs and total costs increase significantly in mastitic cows compared to tolerant ones; additionally, Wolfova et al. [80] reported that the losses of mastitis were owed to the direct financial losses including losses from cost of drugs treatment, service cost, veterinary service, herdsman’s time, the cost for an extra milking machine, losses of the reduced amount of milk and losses of discarded milk during the treatment course.

## 5. Conclusions

Our findings highlight the significance of SNPs in *SELL*, *ABCG2*, *SLC11A1*, *FEZL*, *SOD1*, *CAT*, *GPX1*, and *AhpC/TSA* genes as genetic markers and predisposing factors for mastitis tolerance/susceptibility in Holstein and Brown Swiss breeds; these findings suggest that variability in these genes could be used as proxy biomarkers for such disorders in dairy European cattle. The variable expression pattern of investigated genes in tolerant and susceptible Holstein and Brown Swiss dairy cows to mastitis could be a reference guide; it could be also a biomarker to follow up the health status of dairy cows and to predict the most susceptible risk time for disease occurrence. Consequently, building up an effective management protocol could be obtained to improve health via good breeding and vaccination regimens. Finally, mastitis had detrimental impacts on economic efficiency in dairy farms due to the reduction of milk yield, milk returns and increase in treatment costs.

## Figures and Tables

**Table 1:** Forward and reverse primer sequence, length of PCR product and annealing temperature for genes used in PCR-DNA sequencing.

Primer	Forward	Reverse	Annealing Temperature (°C)	Length of PCR Product (bp)	Reference
*SELL*	5’-GAAAGAAAGTAAGCCTTTCTGG -3′	5′-CCAGAAAGGCTTACTTTCTTTC-3′	60	809	Current study
*ABCG2*	5’-AAAGCTTGCGAAGTGAGGCTGA-3′	5′-GTAATAAGCTCCATTGCAATAC-3′	62	756	Current study
*SLC11A1*	5′-GCTTGCCATGCCCGTGAGGGGCT-3′	5′-TAGTAGAGATGGCAGACCTCGC-3′	64	450	Current study
*FEZL*	5’-GATTGGACCGTCTCAATTATACA-3′	5′-CTGTGTGTTGAGGAGACCGGAC-3′	62	813	Current study
*SOD1*	5′-GCTTGCCATGCCCGTGAGGGGCT-3′	5′-GAATCCAGCCACAGCCCCAGC-3′	60	334	Current study
*CAT*	5′-CTATCCTGACACTCACCGCCAC-3′	5′-GAAAGTCCGCACCTGAGTGACAT-3′	64	268	Current study
*GPX1*	5′-GGTCGCCCGCCTTTTAAAAGCAG-3′	5′-TCGGTCATGAGAGCAGTGGCG-3′	64	534	Current study
*AhpC/TSA*	5′-TAAGAATTGTTTAAACTGAAA-3′	5′-TATGATTCAGCAGTTTTAAGTC-3′	62	480	Current study

-SELL—Selectin L; ABCG2—ATP binding cassette sub-family G member 2; SLC11A1—solute carrier family 11 A1; FEZL—Forebrain Embryonic Zinc Finger-Like; SOD1—Superoxide dismutase 1; CAT; Catalase; GPX1—Glutathione peroxidase 1; and AhpC/TSA—alkyl hydroperoxide reductase/thiol-specific antioxidant.

**Table 2:** Oligonucleotide primers sequence, accession number, annealing temperature and PCR product size of genes used in real-time PCR.

Gene	Primer	Product Length (bp)	Annealing Temperature (°C)	Accession Number	Source
*SELL*	F5′-CAACAGGAAGAGTAAGGAGGAC-3 R5′-TTGTCCATGGCCGCTGCATGAC-3′	151	60	NM_174182.1	Current study
*ABCG2*	F5′-CTGAAGGAGCTGTGTTAAGT-3′ R5′-CCAGAATGGCATTGAGGCCAG-3′	144	62	EU570105.1	Current study
*SLC11A1*	F5′-TGTGGCTGGATTCAAACTGCTC-3′ R5′-AGATGGCAGACCTCGCCCAAGT-3′	123	62	NM_174652.2	Current study
*FEZL*	F5′-CGTGTGCTGCAAGGCCGAGCTG-3′ R5′-GCGGAGTCCAGGTAGTTGAAGTA-3′	138	62	NM_001038198.2	Current study
*SOD1*	F5′-GGAAGCTGTGGGCCTTCACGG-3′ R5′-CCAGCCTGAAGATCCGACTCA-3′	88	64	NM_174615.2	Current study
*CAT*	F5′-TATCCTGACACTCACCGCCA-3′ R5′-CGCTGGTAGTTGGCCACTCGA-3′	92	62	MK423993.1	Current study
*GPX1*	F5′-CTGGATTCGGAAACGGATACC-3′ R5′-ACGTTCTCAATGAGCAGCACCT-3′	164	60	NM_174076.3	Current study
*AhpC/TSA*	F5′-TCTGAATCTATTTTCATGTGTA-3′ R5′-CCACCAATGTTTCCTTACTTA-3′	124	62	XM_005210409.4	Current study
*ß. actin*	F5′-CTAGGCACCAGGGCGTAATG-3′ R5′-CCGTGCTCAATGGGGTACTT-3′	109	60	AF191490.1	Current study

-SELL—Selectin L; ABCG2—ATP binding cassette sub-family G member 2; SLC11A1—solute carrier family 11 A1; FEZL—Forebrain Embryonic Zinc Finger-Like; SOD1—Superoxide dismutase 1; CAT; Catalase; GPX1—Glutathione peroxidase 1; and AhpC/TSA—alkyl hydroperoxide reductase/thiol-specifc antioxidant.

**Table 3:** Distribution of SNPs, type of mutation in investigated genes for mastitis tolerant and affected Holstein and Brown Swiss dairy cows.

Gene	SNPs	Tolerant *n* = 120		Mastitic *n* = 120		Total	Type of Mutation	Amino Acid Number and Type	Chi Value	*p*-Value
		Holstein *n* = 60	Brown Swiss *n* = 60	Holstein *n* = 60	Brown Swiss *n* = 60					
*SELL*	A226G	34	-	-		34/240	Non-synonymous	77 M to V	21.26	<0.0001
	C260T	41	-	-		41/240	Non-synonymous	87 P to L	25.64	<0.0001
	G338A	-	38	-		38/240	Non-synonymous	113 R to K	23.76	<0.0001
	T695C	-		28	-	28/240	Non-synonymous	232 L to P	17.51	<0.0001
*ABCG2*	A91G	-		-	18	18/240	Non-synonymous	31 T o A	11.26	<0.0001
	T108G	-		32		32/240	Non-synonymous	36 H to Q	20.01	<0.0001
	G630A	-	38	-		38/240	Synonymous	210 T	23.76	<0.0001
*SLC11A1*	A160 G	-	33	-		33/240	Non-synonymous	54 T to A	20.63	<0.0001
	A218G	-	29	-		29/240	Non-synonymous	73 Y to C	18.13	<0.0001
	A230C	-		23	-	23/240	Non-synonymous	77 E to A	14.38	<0.0001
*FEZL*	T262A	22	31	-		53/240	Non-synonymous	88 C to N	33.14	<0.0001
	G263A	-	29	-	-	29/240			18.13	<0.0001
	T760C	27	-	-		27/240	Non-synonymous	254 S to P	16.88	<0.0001
*SOD1*	G88A	-		42	-	42/240	Non-synonymous	30 G to R	26.26	<0.0001
	A160G	26	38	-		64/240	Non-synonymous	54 T to A	40.02	<0.0001
	A218G	-	33	-		33/240	Non-synonymous	73 Y to C	20.63	<0.0001
	A230C	-	28	-		28/240	Non-synonymous	77 E to A	17.51	<0.0001
*CAT*	C202T	-		36	23	59/240	Non-synonymous	68 L to F	36.89	<0.0001
*GPX1*	G33A	-		19	-	19/240	Synonymous	11 P	11.88	<0.0001
	T375C	-		27		27/240	Synonymous	125 P	16.88	<0.0001
*AhpC/TSA*	G256T	29	41	-		70/240	Non-synonymous	86 V to F	43.77	<0.0001
	A298G	-	27	-		27/240	Non-synonymous	100 K to E	16.88	<0.0001

-SELL—Selectin L; ABCG2—ATP binding cassette sub-family G member 2; SLC11A1—solute carrier family 11 A1; FEZL—Forebrain Embryonic Zinc Finger-Like; SOD1—Superoxide dismutase 1; CAT; Catalase; GPX1—Glutathione peroxidase 1; and AhpC/TSA—alkyl hydroperoxide reductase/thiol-specifc antioxidant. -A—Alanine; C—Cisteine; E—Glutamic acid; F—Phenylalanine; G—Glycine; H—Histidine; K=Lysine; L—Leucine; M—Methionine; N—Asparagine; P—Proline; Q—Glutamine; R—Argnine; S—Serine; T—Threonine; V—Valine; and Y—Tyrosine.

**Table 4:** Service cost, treatment cost, total veterinary management cost, total cost, total return, net return and reduction in net profit percentage per cow in Holstein and Brown Swiss breeds.

Economic Parameters	Holstein *n* = 120		Brown Swiss *n* = 120	
	Tolerant *n* = 60	Mastitic *n* = 60	Tolerant *n* = 60	Mastitic *n* = 60
Service cost (EGP)	197.30 ± 9.15 ^b^	445.60 ± 7.58 ^a^	203.40 ± 8.47 ^b^	415.20 ± 11.75 ^a^
Treatment cost (EGP)	-	480.65± 8.46	-	395.84 ± 13.90
Veterinary management cost (EGP)	690.50 ± 9.45 ^b^	850.38 ± 14.59 ^a^	670.73 ± 13.98 ^b^	795.35 ± 17.65 ^a^
Total cost (EGP)	39835.24 ± 199.79 ^b^	40978.56 ± 263.82 ^a^	36750.53 ± 220.29 ^b^	38367.78 ± 264.18 ^a^
Total return (EGP)	68579.20 ± 219.26 ^a^	58534.90 ± 128.55 ^c^	59341.10 ± 123.59 ^b^	54757.18 ± 139.51 ^c^
Net Return (EGP)	28743.96 ± 157.19 ^a^	17556.34 ± 113.75 ^d^	22590.57 ± 189.42 ^b^	16389.40 ± 120.36 ^c^
Reduction % in Net profit	-	39	-	27

- a, b, c, d Means of tolerant and mastitic cows within the same row having different upper-case superscripts are significantly different at *p* ≤ 0.05.

## Data Availability

The data that support the findings of this study are available from the corresponding author upon reasonable request.

## Supplementary Materials

The following supporting information can be downloaded at: https://www.mdpi.com/article/10.3390/vetsci9060294/s1.
