# Supplementary material for: Single Nucleotide Polymorphisms, Gene Expression and Economic Evaluation of Parameters Associated with Mastitis Susceptibility in European Cattle Breeds
Source: Vet Sci. 2022 Jun 14;9(6):294. doi: 10.3390/vetsci9060294 (PMC9229636; doi:10.3390/vetsci9060294)
Supplement: Supplementary file 1 [file vetsci-09-00294-s001.zip › vetsci-1759316-supplementary.pdf]

```
TH              GAAAGAAAGTAAGCCTTTCTGGGGACAGTGAGTGCAGTCTCCCTGCACACCCAGCCTTGG      60
NM_174182.1     GAAAGAAAGTAAGCCTTTCTGGGGACAGTGAGTGCAGTCTCCCTGCACACCCAGCCTTGG      60
MH              GAAAGAAAGTAAGCCTTTCTGGGGACAGTGAGTGCAGTCTCCCTGCACACCCAGCCTTGG      60
TB              GAAAGAAAGTAAGCCTTTCTGGGGACAGTGAGTGCAGTCTCCCTGCACACCCAGCCTTGG      60
MB              GAAAGAAAGTAAGCCTTTCTGGGGACAGTGAGTGCAGTCTCCCTGCACACCCAGCCTTGG
                ************************************************************

TH              GCAAGGATCCAGAGACCTGTGGTGCTGAGCTGAGAGGCTGAAGGGCTACGAAAGGACGGA      120
NM_174182.1     GCAAGGATCCAGAGACCTGTGGTGCTGAGCTGAGAGGCTGAAGGGCTACGAAAGGACGGA      120
MH              GCAAGGATCCAGAGACCTGTGGTGCTGAGCTGAGAGGCTGAAGGGCTACGAAAGGACGGA      120
TB              GCAAGGATCCAGAGACCTGTGGTGCTGAGCTGAGAGGCTGAAGGGCTACGAAAGGACGGA      120
MB              GCAAGGATCCAGAGACCTGTGGTGCTGAGCTGAGAGGCTGAAGGGCTACGAAAGGACGGA      120
                ************************************************************

TH              GCAAAGCCATGCTATGTCCATGGAAATGTCAAAATGCTCAGAGGGGCTTATGGAATGTCT      180
NM_174182.1     GCAAAGCCATGCTATGTCCATGGAAATGTCAAAATGCTCAGAGGGGCTTATGGAATGTCT      180
MH              GCAAAGCCATGCTATGTCCATGGAAATGTCAAAATGCTCAGAGGGGCTTATGGAATGTCT      180
TB              GCAAAGCCATGCTATGTCCATGGAAATGTCAAAATGCTCAGAGGGGCTTATGGAATGTCT      180
MB              GCAAAGCCATGCTATGTCCATGGAAATGTCAAAATGCTCAGAGGGGCTTATGGAATGTCT      180
                ************************************************************

TH              TTAAACTGTGGGTCTGGATTATGCTCTGTTGTGATTTCTTTGCTCATCGTGGCACCGATT      240
NM_174182.1     TTAAACTGTGGGTCTGGATTATGCTCTGTTGTGATTTCTTTGCTCATCATGGCACCGATT      240
MH              TTAAACTGTGGGTCTGGATTATGCTCTGTTGTGATTTCTTTGCTCATCATGGCACCGATT      240
TB              TTAAACTGTGGGTCTGGATTATGCTCTGTTGTGATTTCTTTGCTCATCATGGCACCGATT      240
MB              TTAAACTGTGGGTCTGGATTATGCTCTGTTGTGATTTCTTTGCTCATCATGGCACCGATT      240
                ************************************************ ***********

TH              GCTGGACTTACCATTATTCTAAAAGACCCATGCCCTGGGAAAAGGCTAGAGCGTTCTGCA      300
NM_174182.1     GCTGGACTTACCATTATTCCAAAAGACCCATGCCCTGGGAAAAGGCTAGAGCGTTCTGCA      300
MH              GCTGGACTTACCATTATTCCAAAAGACCCATGCCCTGGGAAAAGGCTAGAGCGTTCTGCA      300
TB              GCTGGACTTACCATTATTCCAAAAGACCCATGCCCTGGGAAAAGGCTAGAGCGTTCTGCA      300
MB              GCTGGACTTACCATTATTCCAAAAGACCCATGCCCTGGGAAAAGGCTAGAGCGTTCTGCA      300
                ****************** *****************************************

TH              GGGAAAATTACACAGATTTAGTTGCCATACAAAACAAGGGAGAGATCGAATACCTGAATA      360
NM_174182.1     GGGAAAATTACACAGATTTAGTTGCCATACAAAACAAGGGAGAGATCGAATACCTGAATA      360
MH              GGGAAAATTACACAGATTTAGTTGCCATACAAAACAAGGGAGAGATCGAATACCTGAATA      360
TB              GGGAAAATTACACAGATTTAGTTGCCATACAAAACAAAGGAGAGATCGAATACCTGAATA      360
MB              GGGAAAATTACACAGATTTAGTTGCCATACAAAACAAGGGAGAGATCGAATACCTGAATA      360
                ************************************* **********************

TH              AGACACTTCCCTTCAGCCGTACTTACTACTGGATTGGAATCCGGAAAGTAGAAGGGGTGT      420
NM_174182.1     AGACACTTCCCTTCAGCCGTACTTACTACTGGATTGGAATCCGGAAAGTAGAAGGGGTGT      420
MH              AGACACTTCCCTTCAGCCGTACTTACTACTGGATTGGAATCCGGAAAGTAGAAGGGGTGT      420
TB              AGACACTTCCCTTCAGCCGTACTTACTACTGGATTGGAATCCGGAAAGTAGAAGGGGTGT      420
MB              AGACACTTCCCTTCAGCCGTACTTACTACTGGATTGGAATCCGGAAAGTAGAAGGGGTGT      420
                ************************************************************

TH              GGACTTGGGTGGGAACCAACAAATCTCTCACTGAAGAAGCAAAGAACTGGGGTGCAGGGG      480
NM_174182.1     GGACTTGGGTGGGAACCAACAAATCTCTCACTGAAGAAGCAAAGAACTGGGGTGCAGGGG      480
MH              GGACTTGGGTGGGAACCAACAAATCTCTCACTGAAGAAGCAAAGAACTGGGGTGCAGGGG      480
TB              GGACTTGGGTGGGAACCAACAAATCTCTCACTGAAGAAGCAAAGAACTGGGGTGCAGGGG      480
MB              GGACTTGGGTGGGAACCAACAAATCTCTCACTGAAGAAGCAAAGAACTGGGGTGCAGGGG      480
                ************************************************************

TH              AGCCCAACAACAGGAAGAGTAAGGAGGACTGTGTGGAAATCTATATCAAGAGGAACAAAG      540
NM_174182.1     AGCCCAACAACAGGAAGAGTAAGGAGGACTGTGTGGAAATCTATATCAAGAGGAACAAAG      540
MH              AGCCCAACAACAGGAAGAGTAAGGAGGACTGTGTGGAAATCTATATCAAGAGGAACAAAG      540
TB              AGCCCAACAACAGGAAGAGTAAGGAGGACTGTGTGGAAATCTATATCAAGAGGAACAAAG      540
MB              AGCCCAACAACAGGAAGAGTAAGGAGGACTGTGTGGAAATCTATATCAAGAGGAACAAAG      540
                ************************************************************

TH              ACTCGGGGAAATGGAATGATGATGCCTGCCACAAAGCAAAGACAGCCCTCTGCTACACAG      600
NM_174182.1     ACTCGGGGAAATGGAATGATGATGCCTGCCACAAAGCAAAGACAGCCCTCTGCTACACAG      600
MH              ACTCGGGGAAATGGAATGATGATGCCTGCCACAAAGCAAAGACAGCCCTCTGCTACACAG      600
TB              ACTCGGGGAAATGGAATGATGATGCCTGCCACAAAGCAAAGACAGCCCTCTGCTACACAG      600
MB              ACTCGGGGAAATGGAATGATGATGCCTGCCACAAAGCAAAGACAGCCCTCTGCTACACAG      600
                ************************************************************

TH              CTTCTTGTAAACCCTGGTCATGCAGCGGCCATGGACAATGTGTGGAAGTCATCAATAATT      660
NM_174182.1     CTTCTTGTAAACCCTGGTCATGCAGCGGCCATGGACAATGTGTGGAAGTCATCAATAATT      660
MH              CTTCTTGTAAACCCTGGTCATGCAGCGGCCATGGACAATGTGTGGAAGTCATCAATAATT      660
TB              CTTCTTGTAAACCCTGGTCATGCAGCGGCCATGGACAATGTGTGGAAGTCATCAATAATT      660
MB              CTTCTTGTAAACCCTGGTCATGCAGCGGCCATGGACAATGTGTGGAAGTCATCAATAATT      660
                ************************************************************

TH              ACACCTGCAACTGTGATTTGGGGTACTACGGGCCTGAGTGTCAGTTCGTGACTCAATGTG      720
NM_174182.1     ACACCTGCAACTGTGATTTGGGGTACTACGGGCCTGAGTGTCAGTTCGTGACTCAATGTG      720
MH              ACACCTGCAACTGTGATTTGGGGTACTACGGGCCCGAGTGTCAGTTCGTGACTCAATGTG      720
TB              ACACCTGCAACTGTGATTTGGGGTACTACGGGCCTGAGTGTCAGTTCGTGACTCAATGTG      720
MB              ACACCTGCAACTGTGATTTGGGGTACTACGGGCCTGAGTGTCAGTTCGTGACTCAATGTG      720
```

```
                 ********************************** ************************
TH               TGCCTTTGGAGGCCCCAAAGCTGGGTACCATGGCCTGCACTCACCCTTTGGGGAACTTCA         780
NM_174182.1      TGCCTTTGGAGGCCCCAAAGCTGGGTACCATGGCCTGCACTCACCCTTTGGGGAACTTCA         780
MH               TGCCTTTGGAGGCCCCAAAGCTGGGTACCATGGCCTGCACTCACCCTTTGGGGAACTTCA         780
TB               TGCCTTTGGAGGCCCCAAAGCTGGGTACCATGGCCTGCACTCACCCTTTGGGGAACTTCA         780
MB               TGCCTTTGGAGGCCCCAAAGCTGGGTACCATGGCCTGCACTCACCCTTTGGGGAACTTCA         780
                 ************************************************************
TH               GCTTCATGTCGCAGTGTGCCTTCAACTGC  809
NM_174182.1      GCTTCATGTCGCAGTGTGCCTTCAACTGC  809
MH               GCTTCATGTCGCAGTGTGCCTTCAACTGC  809
TB               GCTTCATGTCGCAGTGTGCCTTCAACTGC  809
MB               GCTTCATGTCGCAGTGTGCCTTCAACTGC  809
                 *****************************
```

Fig. S1. Representative DNA sequence alignment of *SELL* gene (809-bp) among tolerant and mastitis affected Holstein and Brown Swiss dairy cows and reference sequence available in the GenBank gb|NM_174182.1|. TH= Tolerant Holstein; MH= Mastitic Holstein; TB= Tolerant Brown Swiss and MB= Mastitic Brown Swiss. The asterisks represent the similarity.

```
EU570105.1      AAAGCTTGCGAAGTGAGGCTGAAGTCGGTGCTGCCTGCGCTCGCTCGTCGGCCCTCGACC 48
TH              AAAGCTTGCGAAGTGAGGCTGAAGTCGGTGCTGCCTGCGCTCGCTCGTCGGCCCTCGACC 48
MH              AAAGCTTGCGAAGTGAGGCTGAAGTCGGTGCTGCCTGCGCTCGCTCGTCGGCCCTCGACC 48
TB              AAAGCTTGCGAAGTGAGGCTGAAGTCGGTGCTGCCTGCGCTCGCTCGTCGGCCCTCGACC 60
MB              AAAGCTTGCGAAGTGAGGCTGAAGTCGGTGCTGCCTGCGCTCGCTCGTCGGCCCTCGACC 60
                ************************************************************

EU570105.1      GCCGGCTCGCCGCCCGCTCTCTCCGACGTGACGGTAACCCGGGGCCATTGCCTTCCCAGG 120
TH              GCCGGCTCGCCGCCCGCTCTCTCCGACGTGACGGTAACCCGGGGCCATTGCCTTCCCAGG 120
MH              GCCGGCTCGCCGCCCGCTCTCTCCGACGTGACGGTAACCCGGGGCCAGTGCCTTCCCAGG 120
TB              GCCGGCTCGCCGCCCGCTCTCTCCGACGTGACGGTAACCCGGGGCCATTGCCTTCCCAGG 120
MB              GCCGGCTCGCCGCCCGCTCTCTCCGACGTGGCGGTAACCCGGGGCCATTGCCTTCCCAGG 120
                ****************************** **************** ************

EU570105.1      TCAGCCGCTGCGCCGGAAGGCGGAAATGCTCAAAATGTCTTCCAATAGTTACGAGGTTTC 180
TH              TCAGCCGCTGCGCCGGAAGGCGGAAATGCTCAAAATGTCTTCCAATAGTTACGAGGTTTC 180
MH              TCAGCCGCTGCGCCGGAAGGCGGAAATGCTCAAAATGTCTTCCAATAGTTACGAGGTTTC 180
TB              TCAGCCGCTGCGCCGGAAGGCGGAAATGCTCAAAATGTCTTCCAATAGTTACGAGGTTTC 180
MB              TCAGCCGCTGCGCCGGAAGGCGGAAATGCTCAAAATGTCTTCCAATAGTTACGAGGTTTC 180
                ************************************************************

EU570105.1      TATCCCAATGTCAAAAAAACTCAACGGCATTCCAGAGACAACCTCTAAGGACCTGCAGAC 240
TH              TATCCCAATGTCAAAAAAACTCAACGGCATTCCAGAGACAACCTCTAAGGACCTGCAGAC 240
MH              TATCCCAATGTCAAAAAAACTCAACGGCATTCCAGAGACAACCTCTAAGGACCTGCAGAC 240
TB              TATCCCAATGTCAAAAAAACTCAACGGCATTCCAGAGACAACCTCTAAGGACCTGCAGAC 240
MB              TATCCCAATGTCAAAAAAACTCAACGGCATTCCAGAGACAACCTCTAAGGACCTGCAGAC 240
                ************************************************************

EU570105.1      ATTAACTGAAGGAGCTGTGTTAAGTTTTCATAACATCTGCTATCGAGTAAAAGTGAAGAC 300
TH              ATTAACTGAAGGAGCTGTGTTAAGTTTTCATAACATCTGCTATCGAGTAAAAGTGAAGAC 300
MH              ATTAACTGAAGGAGCTGTGTTAAGTTTTCATAACATCTGCTATCGAGTAAAAGTGAAGAC 300
TB              ATTAACTGAAGGAGCTGTGTTAAGTTTTCATAACATCTGCTATCGAGTAAAAGTGAAGAC 300
MB              ATTAACTGAAGGAGCTGTGTTAAGTTTTCATAACATCTGCTATCGAGTAAAAGTGAAGAC 300
                ************************************************************

EU570105.1      TGGCTTTCTACTTTGTCGGAAAACAATTGAGAAAGAAATACTAGCAAATATCAATGGAGT 360
TH              TGGCTTTCTACTTTGTCGGAAAACAATTGAGAAAGAAATACTAGCAAATATCAATGGAGT 360
MH              TGGCTTTCTACTTTGTCGGAAAACAATTGAGAAAGAAATACTAGCAAATATCAATGGAGT 360
TB              TGGCTTTCTACTTTGTCGGAAAACAATTGAGAAAGAAATACTAGCAAATATCAATGGAGT 360
MB              TGGCTTTCTACTTTGTCGGAAAACAATTGAGAAAGAAATACTAGCAAATATCAATGGAGT 360
                ************************************************************

EU570105.1      CATGAAACCTGGCCTCAATGCCATTCTGGGACCCACAGGTGGAGGCAAATCTTCGTTGTT 420
TH              CATGAAACCTGGCCTCAATGCCATTCTGGGACCCACAGGTGGAGGCAAATCTTCGTTGTT 420
MH              CATGAAACCTGGCCTCAATGCCATTCTGGGACCCACAGGTGGAGGCAAATCTTCGTTGTT 420
TB              CATGAAACCTGGCCTCAATGCCATTCTGGGACCCACAGGTGGAGGCAAATCTTCGTTGTT 420
MB              CATGAAACCTGGCCTCAATGCCATTCTGGGACCCACAGGTGGAGGCAAATCTTCGTTGTT 420
                ************************************************************

EU570105.1      AGATATCTTAGCTGCAAGGAAGGATCCACATGGATTATCTGGAGATGTTTTGATCAATGG 480
TH              AGATATCTTAGCTGCAAGGAAGGATCCACATGGATTATCTGGAGATGTTTTGATCAATGG 480
MH              AGATATCTTAGCTGCAAGGAAGGATCCACATGGATTATCTGGAGATGTTTTGATCAATGG 480
TB              AGATATCTTAGCTGCAAGGAAGGATCCACATGGATTATCTGGAGATGTTTTGATCAATGG 480
MB              AGATATCTTAGCTGCAAGGAAGGATCCACATGGATTATCTGGAGATGTTTTGATCAATGG 480
                ************************************************************

EU570105.1      AGCACCTCGACCTGCCAATTTTAAATGTAACTCAGGTTATGTGGTACAAGATGATGTTGT 540
TH              AGCACCTCGACCTGCCAATTTTAAATGTAACTCAGGTTATGTGGTACAAGATGATGTTGT 540
MH              AGCACCTCGACCTGCCAATTTTAAATGTAACTCAGGTTATGTGGTACAAGATGATGTTGT 540
TB              AGCACCTCGACCTGCCAATTTTAAATGTAACTCAGGTTATGTGGTACAAGATGATGTTGT 540
MB              AGCACCTCGACCTGCCAATTTTAAATGTAACTCAGGTTATGTGGTACAAGATGATGTTGT 540
                ************************************************************

EU570105.1      GATGGGAACTCTGACAGTGAGAGAAAACTTACAGTTCTCAGCAGCCCTTCGGCTTCCAAC 600
TH              GATGGGAACTCTGACAGTGAGAGAAAACTTACAGTTCTCAGCAGCCCTTCGGCTTCCAAC 600
MH              GATGGGAACTCTGACAGTGAGAGAAAACTTACAGTTCTCAGCAGCCCTTCGGCTTCCAAC 600
TB              GATGGGAACTCTGACAGTGAGAGAAAACTTACAGTTCTCAGCAGCCCTTCGGCTTCCAAC 600
MB              GATGGGAACTCTGACAGTGAGAGAAAACTTACAGTTCTCAGCAGCCCTTCGGCTTCCAAC 600
                ************************************************************

EU570105.1      AACTATGACAAGTTACGAAAAAAATGAACGGATTAACAAGGTTATTCAAGAGTTAGGTCT 660
TH              AACTATGACAAGTTACGAAAAAAATGAACGGATTAACAAGGTTATTCAAGAGTTAGGTCT 660
MH              AACTATGACAAGTTACGAAAAAAATGAACGGATTAACAAGGTTATTCAAGAGTTAGGTCT 660
```

```
TB              AACTATGACAAGTTACGAAAAAAATGAACAGATTAACAAGGTTATTCAAGAGTTAGGTCT 660
MB              AACTATGACAAGTTACGAAAAAAATGAACGGATTAACAAGGTTATTCAAGAGTTAGGTCT 660
                ***************************** ******************************

EU570105.1      GGATAAAGTGGCAGATTCCAAGGTTGGAACTCAGTTTATCCGTGGTGTGTCTGGAGGAGA 720
TH              GGATAAAGTGGCAGATTCCAAGGTTGGAACTCAGTTTATCCGTGGTGTGTCTGGAGGAGA 720
MH              GGATAAAGTGGCAGATTCCAAGGTTGGAACTCAGTTTATCCGTGGTGTGTCTGGAGGAGA 720
TB              GGATAAAGTGGCAGATTCCAAGGTTGGAACTCAGTTTATCCGTGGTGTGTCTGGAGGAGA 720
MB              GGATAAAGTGGCAGATTCCAAGGTTGGAACTCAGTTTATCCGTGGTGTGTCTGGAGGAGA 720
                ************************************************************

EU570105.1      AAGAAAAAGGACTAGTATTGCAATGGAGCTTATTAC        756
TH              AAGAAAAAGGACTAGTATTGCAATGGAGCTTATTAC        756
MH              AAGAAAAAGGACTAGTATTGCAATGGAGCTTATTAC        756
TB              AAGAAAAAGGACTAGTATTGCAATGGAGCTTATTAC        756
MB              AAGAAAAAGGACTAGTATTGCAATGGAGCTTATTAC        756
                ************************************
```

Fig. S2. Representative DNA sequence alignment of *ABCG2* gene (756-bp) among tolerant and mastitis affected Holstein and Brown Swiss dairy cows and reference sequence available in the GenBank gb|EU570105.1|. TH= Tolerant Holstein; MH= Mastitic Holstein; TB= Tolerant Brown Swiss and MB= Mastitic Brown Swiss. The asterisks represent the similarity.

```
NM_174652.2      GCTTGCCATGCCCGTGAGGGGCTGCCCGGCACGCCAGCCACTCGCACAGAGAGTGCCCGA         60
TH               GCTTGCCATGCCCGTGAGGGGCTGCCCGGCACGCCAGCCACTCGCACAGAGAGTGCCCGA         60
MH               GCTTGCCATGCCCGTGAGGGGCTGCCCGGCACGCCAGCCACTCGCACAGAGAGTGCCCGA         60
TB               GCTTGCCATGCCCGTGAGGGGCTGCCCGGCACGCCAGCCACTCGCACAGAGAGTGCCCGA         60
MB               GCTTGCCATGCCCGTGAGGGGCTGCCCGGCACGCCAGCCACTCGCACAGAGAGTGCCCGA         60
                 ************************************************************

NM_174652.2      GCCTGCGGTCCTCATGTCAGGTGACACGGGCCCCCCAAAGCAGGGAGGGACCAGATATGG         120
TH               GCCTGCGGTCCTCATGTCAGGTGACACGGGCCCCCCAAAGCAGGGAGGGACCAGATATGG         120
MH               GCCTGCGGTCCTCATGTCAGGTGACACGGGCCCCCCAAAGCAGGGAGGGACCAGATATGG         120
TB               GCCTGCGGTCCTCATGTCAGGTGACACGGGCCCCCCAAAGCAGGGAGGGACCAGATATGG         120
MB               GCCTGCGGTCCTCATGTCAGGTGACACGGGCCCCCCAAAGCAGGGAGGGACCAGATATGG         120
                 ************************************************************

NM_174652.2      CTCCATCTCCAGCCCACCCAGTCCAGAGCCACAGCAAGCACCTCCCGGAGGGACCTACCT         180
TH               CTCCATCTCCAGCCCACCCAGTCCAGAGCCACAGCAAGCACCTCCCGGAGGGACCTACCT         180
MH               CTCCATCTCCAGCCCACCCAGTCCAGAGCCACAGCAAGCACCTCCCGGAGGGACCTACCT         180
TB               CTCCATCTCCAGCCCACCCAGTCCAGAGCCACAGCAAGCGCCTCCCGGAGGGACCTACCT         180
MB               CTCCATCTCCAGCCCACCCAGTCCAGAGCCACAGCAAGCACCTCCCGGAGGGACCTACCT         180
                 *************************************** ********************

NM_174652.2      AAGTGAGAAGATCCCCATTCCGGATACAGAATCGGGTACATTCAGCCTGAGGAAGCTGTG         240
TH               AAGTGAGAAGATCCCCATTCCGGATACAGAATCGGGTACATTCAGCCTGAGGAAGCTGTG         240
MH               AAGTGAGAAGATCCCCATTCCGGATACAGAATCGGGTACATTCAGCCTGCGGAAGCTGTG         240
TB               AAGTGAGAAGATCCCCATTCCGGATACAGAATCGGGTGCATTCAGCCTGAGGAAGCTGTG         240
MB               AAGTGAGAAGATCCCCATTCCGGATACAGAATCGGGTACATTCAGCCTGAGGAAGCTGTG         240
                 ************************************* *********** **********

NM_174652.2      GGCCTTCACGGGGCCTGGATTCCTCATGAGCATCGCATTCCTGGACCCAGGAAACATTGA         300
TH               GGCCTTCACGGGGCCTGGATTCCTCATGAGCATCGCATTCCTGGACCCAGGAAACATTGA         300
MH               GGCCTTCACGGGGCCTGGATTCCTCATGAGCATCGCATTCCTGGACCCAGGAAACATTGA         300
TB               GGCCTTCACGGGGCCTGGATTCCTCATGAGCATCGCATTCCTGGACCCAGGAAACATTGA         300
MB               GGCCTTCACGGGGCCTGGATTCCTCATGAGCATCGCATTCCTGGACCCAGGAAACATTGA         300
                 ************************************************************

NM_174652.2      GTCGGATCTTCAGGCTGGGGCTGTGGCTGGATTCAAACTGCTCTGGGTGCTGCTGTGGGC         360
TH               GTCGGATCTTCAGGCTGGGGCTGTGGCTGGATTCAAACTGCTCTGGGTGCTGCTGTGGGC         360
MH               GTCGGATCTTCAGGCTGGGGCTGTGGCTGGATTCAAACTGCTCTGGGTGCTGCTGTGGGC         360
TB               GTCGGATCTTCAGGCTGGGGCTGTGGCTGGATTCAAACTGCTCTGGGTGCTGCTGTGGGC         360
MB               GTCGGATCTTCAGGCTGGGGCTGTGGCTGGATTCAAACTGCTCTGGGTGCTGCTGTGGGC         360
                 ************************************************************

NM_174652.2      CACAGTGTTGGGCTTGCTTTGCCAGCGACTGGCTGCCCGGCTGGGCGTGGTGACAGGCAA         420
TH               CACAGTGTTGGGCTTGCTTTGCCAGCGACTGGCTGCCCGGCTGGGCGTGGTGACAGGCAA         420
MH               CACAGTGTTGGGCTTGCTTTGCCAGCGACTGGCTGCCCGGCTGGGCGTGGTGACAGGCAA         420
TB               CACAGTGTTGGGCTTGCTTTGCCAGCGACTGGCTGCCCGGCTGGGCGTGGTGACAGGCAA         420
MB               CACAGTGTTGGGCTTGCTTTGCCAGCGACTGGCTGCCCGGCTGGGCGTGGTGACAGGCAA         420
                 ************************************************************

NM_174652.2      GGACTTGGGCGAGGTCTGCCATCTCTACTA 450
TH               GGACTTGGGCGAGGTCTGCCATCTCTACTA 450
MH               GGACTTGGGCGAGGTCTGCCATCTCTACTA 450
TB               GGACTTGGGCGAGGTCTGCCATCTCTACTA 450
MB               GGACTTGGGCGAGGTCTGCCATCTCTACTA 450
                 ******************************
```

Fig. S3. Representative DNA sequence alignment of *SLC11A1* gene (450-bp) among tolerant and mastitis affected Holstein and Brown Swiss dairy cows and reference sequence available in the GenBank gb|NM_174652.2|. TH= Tolerant Holstein; MH= Mastitic Holstein; TB= Tolerant Brown Swiss and MB= Mastitic Brown Swiss. The asterisks represent the similarity.

```
NM_001038198.2      GATTGGACCGTCTCAATTATACAAACATGACTTCATGTGATGTCAGCTGAATGTAAAAGA      60
TH                  GATTGGACCGTCTCAATTATACAAACATGACTTCATGTGATGTCAGCTGAATGTAAAAGA      60
MH                  GATTGGACCGTCTCAATTATACAAACATGACTTCATGTGATGTCAGCTGAATGTAAAAGA      60
TB                  GATTGGACCGTCTCAATTATACAAACATGACTTCATGTGATGTCAGCTGAATGTAAAAGA      60
MB                  GATTGGACCGTCTCAATTATACAAACATGACTTCATGTGATGTCAGCTGAATGTAAAAGA      60
                    ************************************************************
NM_001038198.2      CAGTGATCTCACGCGGAGGGGAAGATGTTTGCCATCAAAATGTGACAGAGGAGACAGGCT      120
TH                  CAGTGATCTCACGCGGAGGGGAAGATGTTTGCCATCAAAATGTGACAGAGGAGACAGGCT      120
MH                  CAGTGATCTCACGCGGAGGGGAAGATGTTTGCCATCAAAATGTGACAGAGGAGACAGGCT      120
TB                  CAGTGATCTCACGCGGAGGGGAAGATGTTTGCCATCAAAATGTGACAGAGGAGACAGGCT      120
MB                  CAGTGATCTCACGCGGAGGGGAAGATGTTTGCCATCAAAATGTGACAGAGGAGACAGGCT      120
                    ************************************************************
NM_001038198.2      GCATGGCTCGGACCGCATCTCCTTGGCGGTGGGGGAAAGAGACTTAGAGGAGAGAGGCTG      180
TH                  GCATGGCTCGGACCGCATCTCCTTGGCGGTGGGGGAAAGAGACTTAGAGGAGAGAGGCTG      180
MH                  GCATGGCTCGGACCGCATCTCCTTGGCGGTGGGGGAAAGAGACTTAGAGGAGAGAGGCTG      180
TB                  GCATGGCTCGGACCGCATCTCCTTGGCGGTGGGGGAAAGAGACTTAGAGGAGAGAGGCTG      180
MB                  GCATGGCTCGGACCGCATCTCCTTGGCGGTGGGGGAAAGAGACTTAGAGGAGAGAGGCTG      180
                    ************************************************************
NM_001038198.2      CGCCCTGGCCCAGTCCGGTACGACTCGGCTCGGCGCGCCATGGCAAGCTCGGCTTCCCTG      240
TH                  CGCCCTGGCCCAGTCCGGTACGACTCGGCTCGGCGCGCCATGGCAAGCTCGGCTTCCCTG      240
MH                  CGCCCTGGCCCAGTCCGGTACGACTCGGCTCGGCGCGCCATGGCAAGCTCGGCTTCCCTG      240
TB                  CGCCCTGGCCCAGTCCGGTACGACTCGGCTCGGCGCGCCATGGCAAGCTCGGCTTCCCTG      240
MB                  CGCCCTGGCCCAGTCCGGTACGACTCGGCTCGGCGCGCCATGGCAAGCTCGGCTTCCCTG      240
                    ************************************************************
NM_001038198.2      GAGACCATGGTGCCCCCGGCCTGCCCGCGCGCAGGAGCGTCGCCGGCCACTTCCAAGACT      300
TH                  GAGACCATGGTGCCCCCGGCCTGCCCGCGCGCAGGAGCGTCGCCGGCCACTTCCAAGACT      300
MH                  GAGACCATGGTGCCCCCGGCCAGCCCGCGCGCAGGAGCGTCGCCGGCCACTTCCAAGACT      300
TB                  GAGACCATGGTGCCCCCGGCCTACCCGCGCGCAGGAGCGTCGCCGGCCACTTCCAAGACT      300
MB                  GAGACCATGGTGCCCCCGGCCAGCCCGCGCGCAGGAGCGTCGCCGGCCACTTCCAAGACT      300
                    *********************  *************************************
NM_001038198.2      CTGGCCTTTTCCATCGAGCGCATCATGGCTAAGACGTCGGAGCCCCGCGCGCCCTTTGAG      360
TH                  CTGGCCTTTTCCATCGAGCGCATCATGGCTAAGACGTCGGAGCCCCGCGCGCCCTTTGAG      360
MH                  CTGGCCTTTTCCATCGAGCGCATCATGGCTAAGACGTCGGAGCCCCGCGCGCCCTTTGAG      360
TB                  CTGGCCTTTTCCATCGAGCGCATCATGGCTAAGACGTCGGAGCCCCGCGCGCCCTTTGAG      360
MB                  CTGGCCTTTTCCATCGAGCGCATCATGGCTAAGACGTCGGAGCCCCGCGCGCCCTTTGAG      360
                    ************************************************************
NM_001038198.2      CCCCGGCCGGGGGCACTGGAGGCGGATAGCGGCCAGGGCAAGAAATTGCTCAACCTCTGC      420
TH                  CCCCGGCCGGGGGCACTGGAGGCGGATAGCGGCCAGGGCAAGAAATTGCTCAACCTCTGC      420
MH                  CCCCGGCCGGGGGCACTGGAGGCGGATAGCGGCCAGGGCAAGAAATTGCTCAACCTCTGC      420
TB                  CCCCGGCCGGGGGCACTGGAGGCGGATAGCGGCCAGGGCAAGAAATTGCTCAACCTCTGC      420
MB                  CCCCGGCCGGGGGCACTGGAGGCGGATAGCGGCCAGGGCAAGAAATTGCTCAACCTCTGC      420
                    ************************************************************
NM_001038198.2      TCGCCGCTGCCCTGTATGATCCCCCTCCAGCCCCTAGGCTACGAGATGCCGTCCAAGACG      480
TH                  TCGCCGCTGCCCTGTATGATCCCCCTCCAGCCCCTAGGCTACGAGATGCCGTCCAAGACG      480
MH                  TCGCCGCTGCCCTGTATGATCCCCCTCCAGCCCCTAGGCTACGAGATGCCGTCCAAGACG      480
TB                  TCGCCGCTGCCCTGTATGATCCCCCTCCAGCCCCTAGGCTACGAGATGCCGTCCAAGACG      480
MB                  TCGCCGCTGCCCTGTATGATCCCCCTCCAGCCCCTAGGCTACGAGATGCCGTCCAAGACG      480
                    ************************************************************
NM_001038198.2      CTGCTCAGTTACTCTGAGCTCTGGAAAAGCAGCCTCCGGGCGGGCGGCAGCGGCGGCGGA      540
TH                  CTGCTCAGTTACTCTGAGCTCTGGAAAAGCAGCCTCCGGGCGGGCGGCAGCGGCGGCGGA      540
MH                  CTGCTCAGTTACTCTGAGCTCTGGAAAAGCAGCCTCCGGGCGGGCGGCAGCGGCGGCGGA      540
TB                  CTGCTCAGTTACTCTGAGCTCTGGAAAAGCAGCCTCCGGGCGGGCGGCAGCGGCGGCGGA      540
MB                  CTGCTCAGTTACTCTGAGCTCTGGAAAAGCAGCCTCCGGGCGGGCGGCAGCGGCGGCGGA      540
                    ************************************************************
NM_001038198.2      GGAGGAGGTGGAGGCGGCGGTGGGGGGGCCCCGGTGTGCGGCGCCAGCGGCTTGTGCAAA      600
TH                  GGAGGAGGTGGAGGCGGCGGTGGGGGGGCCCCGGTGTGCGGCGCCAGCGGCTTGTGCAAA      600
MH                  GGAGGAGGTGGAGGCGGCGGTGGGGGGGCCCCGGTGTGCGGCGCCAGCGGCTTGTGCAAA      600
TB                  GGAGGAGGTGGAGGCGGCGGTGGGGGGGCCCCGGTGTGCGGCGCCAGCGGCTTGTGCAAA      600
MB                  GGAGGAGGTGGAGGCGGCGGTGGGGGGGCCCCGGTGTGCGGCGCCAGCGGCTTGTGCAAA      600
                    ************************************************************
NM_001038198.2      ACCAACTGTGGCGTGTGCTGCAAGGCCGAGCTGGGCCTGGTACCGTCGGCGCTGCCCGCG      660
TH                  ACCAACTGTGGCGTGTGCTGCAAGGCCGAGCTGGGCCTGGTACCGTCGGCGCTGCCCGCG      660
MH                  ACCAACTGTGGCGTGTGCTGCAAGGCCGAGCTGGGCCTGGTACCGTCGGCGCTGCCCGCG      660
TB                  ACCAACTGTGGCGTGTGCTGCAAGGCCGAGCTGGGCCTGGTACCGTCGGCGCTGCCCGCG      660
MB                  ACCAACTGTGGCGTGTGCTGCAAGGCCGAGCTGGGCCTGGTACCGTCGGCGCTGCCCGCG      660
                    ************************************************************
NM_001038198.2      GGCAGGGTCATCAAGCCGCAGGTCATCAACCAGGCTGTGGGGCTTCCGGCCAGCGGTTCG      720
TH                  GGCAGGGTCATCAAGCCGCAGGTCATCAACCAGGCTGTGGGGCTTCCGGCCAGCGGTTCG      720
MH                  GGCAGGGTCATCAAGCCGCAGGTCATCAACCAGGCTGTGGGGCTTCCGGCCAGCGGTTCG      720
TB                  GGCAGGGTCATCAAGCCGCAGGTCATCAACCAGGCTGTGGGGCTTCCGGCCAGCGGTTCG      720
MB                  GGCAGGGTCATCAAGCCGCAGGTCATCAACCAGGCTGTGGGGCTTCCGGCCAGCGGTTCG      720
```

```
                     ************************************************************
NM_001038198.2       CTCTACTACTTCAACTACCTGGACTCCGCCACTTACCCGTCGTCTGAGCTCCTCGGCGGC      780
TH                   CTCTACTACTTCAACTACCTGGACTCCGCCACTTACCCGTCGTCTGAGCTCCTCGGCGGC      780
MH                   CTCTACTACTTCAACTACCTGGACTCCGCCACTTACCCGCCGTCTGAGCTCCTCGGCGGC      780
TB                   CTCTACTACTTCAACTACCTGGACTCCGCCACTTACCCGCCGTCTGAGCTCCTCGGCGGC      780
MB                   CTCTACTACTTCAACTACCTGGACTCCGCCACTTACCCGCCGTCTGAGCTCCTCGGCGGC      780
                     *************************************** ********************
NM_001038198.2       CACCTCTTCCCGTCCGGTCTCCTCAACACACAG 813
TH                   CACCTCTTCCCGTCCGGTCTCCTCAACACACAG 813
MH                   CACCTCTTCCCGTCCGGTCTCCTCAACACACAG 813
TB                   CACCTCTTCCCGTCCGGTCTCCTCAACACACAG 813
MB                   CACCTCTTCCCGTCCGGTCTCCTCAACACACAG 813
                     *********************************
```

Fig. S4. Representative DNA sequence alignment of *FEZL* gene (813-bp) among tolerant and mastitis affected Holstein and Brown Swiss dairy cows and reference sequence available in the GenBank gb|NM_001038198.2|. TH= Tolerant Holstein; MH= Mastitic Holstein; TB= Tolerant Brown Swiss and MB= Mastitic Brown Swiss. The asterisks represent the similarity.

```
NM_174615.2      GCTTGCCATGCCCGTGAGGGGCTGCCCGGCACGCCAGCCACTCGCACAGAGAGTGCCCGA         60
TH               GCTTGCCATGCCCGTGAGGGGCTGCCCGGCACGCCAGCCACTCGCACAGAGAGTGCCCGA         60
MH               GCTTGCCATGCCCGTGAGGGGCTGCCCGGCACGCCAGCCACTCGCACAGAGAGTGCCCGA         60
TB               GCTTGCCATGCCCGTGAGGGGCTGCCCGGCACGCCAGCCACTCGCACAGAGAGTGCCCGA         60
MB               GCTTGCCATGCCCGTGAGGGGCTGCCCGGCACGCCAGCCACTCGCACAGAGAGTGCCCGA         60
                 ************************************************************

NM_174615.2      GCCTGCGGTCCTCATGTCAGGTGACACGGGCCCCCCAAAGCAGGGAGGGACCAGATATGG         120
TH               GCCTGCGGTCCTCATGTCAGGTGACACAGGCCCCCCAAAGCAGGGAGGGACCAGATATGG         120
MH               GCCTGCGGTCCTCATGTCAGGTGACACGGGCCCCCCAAAGCAGGGAGGGACCAGATATGG         120
TB               GCCTGCGGTCCTCATGTCAGGTGACACAGGCCCCCCAAAGCAGGGAGGGACCAGATATGG         120
MB               GCCTGCGGTCCTCATGTCAGGTGACACAGGCCCCCCAAAGCAGGGAGGGACCAGATATGG         120
                 *************************** ********************************

NM_174615.2      CTCCATCTCCAGCCCACCCAGTCCAGAGCCACAGCAAGCACCTCCCGGAGGGACCTACCT         180
TH               CTCCATCTCCAGCCCACCCAGTCCAGAGCCACAGCAAGCGCCTCCCGGAGGGACCTACCT         180
MH               CTCCATCTCCAGCCCACCCAGTCCAGAGCCACAGCAAGCACCTCCCGGAGGGACCTACCT         180
TB               CTCCATCTCCAGCCCACCCAGTCCAGAGCCACAGCAAGCGCCTCCCGGAGGGACCTACCT         180
MB               CTCCATCTCCAGCCCACCCAGTCCAGAGCCACAGCAAGCACCTCCCGGAGGGACCTACCT         180
                 *************************************** ********************

NM_174615.2      AAGTGAGAAGATCCCCATTCCGGATACAGAATCGGGTACATTCAGCCTGAGGAAGCTGTG         240
TH               AAGTGAGAAGATCCCCATTCCGGATACAGAATCGGGTACATTCAGCCTGAGGAAGCTGTG         240
MH               AAGTGAGAAGATCCCCATTCCGGATACAGAATCGGGTACATTCAGCCTGAGGAAGCTGTG         240
TB               AAGTGAGAAGATCCCCATTCCGGATACAGAATCGGGTGCATTCAGCCTGCGGAAGCTGTG         240
MB               AAGTGAGAAGATCCCCATTCCGGATACAGAATCGGGTACATTCAGCCTGAGGAAGCTGTG         240
                 ************************************* *********** **********

NM_174615.2      GGCCTTCACGGGGCCTGGATTCCTCATGAGCATCGCATTCCTGGACCCAGGAAACATTGA         300
TH               GGCCTTCACGGGGCCTGGATTCCTCATGAGCATCGCATTCCTGGACCCAGGAAACATTGA         300
MH               GGCCTTCACGGGGCCTGGATTCCTCATGAGCATCGCATTCCTGGACCCAGGAAACATTGA         300
TB               GGCCTTCACGGGGCCTGGATTCCTCATGAGCATCGCATTCCTGGACCCAGGAAACATTGA         300
MB               GGCCTTCACGGGGCCTGGATTCCTCATGAGCATCGCATTCCTGGACCCAGGAAACATTGA         300
                 ************************************************************

NM_174615.2      GTCGGATCTTCAGGCTGGGGCTGTGGCTGGATTC      334
TH               GTCGGATCTTCAGGCTGGGGCTGTGGCTGGATTC      334
MH               GTCGGATCTTCAGGCTGGGGCTGTGGCTGGATTC      334
TB               GTCGGATCTTCAGGCTGGGGCTGTGGCTGGATTC      334
MB               GTCGGATCTTCAGGCTGGGGCTGTGGCTGGATTC      334
                 **********************************
```

Fig. S5. Representative DNA sequence alignment of *SOD1* gene (334-bp) among tolerant and mastitis affected Holstein and Brown Swiss dairy cows and reference sequence available in the GenBank gb|NM_174615.2|. TH= Tolerant Holstein; MH= Mastitic Holstein; TB= Tolerant Brown Swiss and MB= Mastitic Brown Swiss. The asterisks represent the similarity.

```
MK423993.1      CTATCCTGACACTCACCGCCACCGCCTGGGACCCAACTATCTCCAGATACCTGTGAACTG 60
TH              CTATCCTGACACTCACCGCCACCGCCTGGGACCCAACTATCTCCAGATACCTGTGAACTG 60
MH              CTATCCTGACACTCACCGCCACCGCCTGGGACCCAACTATCTCCAGATACCTGTGAACTG 60
TB              CTATCCTGACACTCACCGCCACCGCCTGGGACCCAACTATCTCCAGATACCTGTGAACTG 60
MB              CTATCCTGACACTCACCGCCACCGCCTGGGACCCAACTATCTCCAGATACCTGTGAACTG 60
                ************************************************************

MK423993.1      TCCCTACCGTGCTCGAGTGGCCAACTACCAGCGTGACGGCCCCATGTGCATGATGGACAA 120
TH              TCCCTACCGTGCTCGAGTGGCCAACTACCAGCGTGACGGCCCCATGTGCATGATGGACAA 120
MB              TCCCTACCGTGCTCGAGTGGCCAACTACCAGCGTGACGGCCCCATGTGCATGATGGACAA 120
TB              TCCCTACCGTGCTCGAGTGGCCAACTACCAGCGTGACGGCCCCATGTGCATGATGGACAA 120
MB              TCCCTACCGTGCTCGAGTGGCCAACTACCAGCGTGACGGCCCCATGTGCATGATGGACAA 120
                ************************************************************

MK423993.1      TCAGGGTGGGGCTCCAAATTACTACCCCAATAGCTTTAGTGCTCCCGAGCATCAGCCTTC 180
TH              TCAGGGTGGGGCTCCAAATTACTACCCCAATAGCTTTAGTGCTCCCGAGCATCAGCCTTC 180
MH              TCAGGGTGGGGCTCCAAATTACTACCCCAATAGCTTTAGTGCTCCCGAGCATCAGCCTTC 180
TB              TCAGGGTGGGGCTCCAAATTACTACCCCAATAGCTTTAGTGCTCCCGAGCATCAGCCTTC 180
MB              TCAGGGTGGGGCTCCAAATTACTACCCCAATAGCTTTAGTGCTCCCGAGCATCAGCCTTC 180
                ************************************************************

MK423993.1      TGCCCTGGAACACAGGACCCACTTCTCTGGGGATGTACAGCGCTTCAACAGTGCCAACGA 240
TH              TGCCCTGGAACACAGGACCCACTTCTCTGGGGATGTACAGCGCTTCAACAGTGCCAACGA 240
MH              TGCCCTGGAACACAGGACCCATTTCTCTGGGGATGTACAGCGCTTCAACAGTGCCAACGA 240
TB              TGCCCTGGAACACAGGACCCACTTCTCTGGGGATGTACAGCGCTTCAACAGTGCCAACGA 240
MB              TGCCCTGGAACACAGGACCCATTTCTCTGGGGATGTACAGCGCTTCAACAGTGCCAACGA 240
                ********************* **************************************

MK423993.1      TGACAATGTCACTCAGGTGCGGACTTTC       268
TH              TGACAATGTCACTCAGGTGCGGACTTTC       268
MH              TGACAATGTCACTCAGGTGCGGACTTTC       268
TB              TGACAATGTCACTCAGGTGCGGACTTTC       268
MB              TGACAATGTCACTCAGGTGCGGACTTTC       268
                ****************************
```

Fig. S6. Representative DNA sequence alignment of *CAT* gene (268-bp) among tolerant and mastitis affected Holstein and Brown Swiss dairy cows and reference sequence available in the GenBank gb|MK423993.1|. TH= Tolerant Holstein; MH= Mastitic Holstein; TB= Tolerant Brown Swiss and MB= Mastitic Brown Swiss. The asterisks represent the similarity.

```
NM_174076.3      GGTCGCCCGCCTTTTAAAAGCAGGCGCTTCCCGACCCCCTTGTTCTTCAAGTCCGCGGCG         60
TH               GGTCGCCCGCCTTTTAAAAGCAGGCGCTTCCCGACCCCCTTGTTCTTCAAGTCCGCGGCG         60
MH               GGTCGCCCGCCTTTTAAAAGCAGGCGCTTCCCAACCCCCTTGTTCTTCAAGTCCGCGGCG         60
TB               GGTCGCCCGCCTTTTAAAAGCAGGCGCTTCCCGACCCCCTTGTTCTTCAAGTCCGCGGCG         60
MB               GGTCGCCCGCCTTTTAAAAGCAGGCGCTTCCCGACCCCCTTGTTCTTCAAGTCCGCGGCG         60
                 ******************************** ***************************

NM_174076.3      CTCTGCTCTGGATTCGGAAACGGATACCATGTGCGCCGCTCAGCGCTCGGCGGCCGCCCT         120
TH               CTCTGCTCTGGATTCGGAAACGGATACCATGTGCGCCGCTCAGCGCTCGGCGGCCGCCCT         120
MH               CTCTGCTCTGGATTCGGAAACGGATACCATGTGCGCCGCTCAGCGCTCGGCGGCCGCCCT         120
TB               CTCTGCTCTGGATTCGGAAACGGATACCATGTGCGCCGCTCAGCGCTCGGCGGCCGCCCT         120
MB               CTCTGCTCTGGATTCGGAAACGGATACCATGTGCGCCGCTCAGCGCTCGGCGGCCGCCCT         120
                 ************************************************************

NM_174076.3      GGCGGCGGCAGCCCCGCGCACAGTGTACGCCTTCTCCGCGCGCCCTCTGGCCGGCGGGGA         180
TH               GGCGGCGGCAGCCCCGCGCACAGTGTACGCCTTCTCCGCGCGCCCTCTGGCCGGCGGGGA         180
MH               GGCGGCGGCAGCCCCGCGCACAGTGTACGCCTTCTCCGCGCGCCCTCTGGCCGGCGGGGA         180
TB               GGCGGCGGCAGCCCCGCGCACAGTGTACGCCTTCTCCGCGCGCCCTCTGGCCGGCGGGGA         180
MB               GGCGGCGGCAGCCCCGCGCACAGTGTACGCCTTCTCCGCGCGCCCTCTGGCCGGCGGGGA         180
                 ************************************************************

NM_174076.3      GCCCTTCAACCTGTCCTCCCTGCGGGGCAAGGTGCTGCTCATTGAGAACGTAGCATCGCT         240
TH               GCCCTTCAACCTGTCCTCCCTGCGGGGCAAGGTGCTGCTCATTGAGAACGTAGCATCGCT         240
MH               GCCCTTCAACCTGTCCTCCCTGCGGGGCAAGGTGCTGCTCATTGAGAACGTAGCATCGCT         240
TB               GCCCTTCAACCTGTCCTCCCTGCGGGGCAAGGTGCTGCTCATTGAGAACGTAGCATCGCT         240
MB               GCCCTTCAACCTGTCCTCCCTGCGGGGCAAGGTGCTGCTCATTGAGAACGTAGCATCGCT         240
                 ************************************************************

NM_174076.3      CTGAGGCACAACGGTGCGGGACTACACCCAGATGAATGACCTGCAGCGGCGCCTTGGACC         300
TH               CTGAGGCACAACGGTGCGGGACTACACCCAGATGAATGACCTGCAGCGGCGCCTTGGACC         300
MH               CTGAGGCACAACGGTGCGGGACTACACCCAGATGAATGACCTGCAGCGGCGCCTTGGACC         300
TB               CTGAGGCACAACGGTGCGGGACTACACCCAGATGAATGACCTGCAGCGGCGCCTTGGACC         300
MB               CTGAGGCACAACGGTGCGGGACTACACCCAGATGAATGACCTGCAGCGGCGCCTTGGACC         300
                 ************************************************************

NM_174076.3      CCGGGGCCTGGTCGTGCTCGGCTTCCCCTGCAACCAGTTTGGGCATCAGGAAAACGCCAA         360
TH               CCGGGGCCTGGTCGTGCTCGGCTTCCCCTGCAACCAGTTTGGGCATCAGGAAAACGCCAA         360
MH               CCGGGGCCTGGTCGTGCTCGGCTTCCCCTGCAACCAGTTTGGGCATCAGGAAAACGCCAA         360
TB               CCGGGGCCTGGTCGTGCTCGGCTTCCCCTGCAACCAGTTTGGGCATCAGGAAAACGCCAA         360
MB               CCGGGGCCTGGTCGTGCTCGGCTTCCCCTGCAACCAGTTTGGGCATCAGGAAAACGCCAA         360
                 ************************************************************

NM_174076.3      GAACGAGGAGATCCTGAATTGCCTGAAGTACGTCCGACCAGGCGGCGGGTTCGAGCCCAA         420
TH               GAACGAGGAGATCCTGAATTGCCTGAAGTACGTCCGACCAGGCGGCGGGTTCGAGCCCAA         420
MH               GAACGAGGAGATCCCGAATTGCCTGAAGTACGTCCGACCAGGCGGCGGGTTCGAGCCCAA         420
TB               GAACGAGGAGATCCTGAATTGCCTGAAGTACGTCCGACCAGGCGGCGGGTTCGAGCCCAA         420
MB               GAACGAGGAGATCCTGAATTGCCTGAAGTACGTCCGACCAGGCGGCGGGTTCGAGCCCAA         420
                 ************** *********************************************

NM_174076.3      CTTTATGCTCTTCGAAAAGTGCGAGGTGAATGGCGAGAAGGCGCATCCGCTCTTCGCCTT         480
TH               CTTTATGCTCTTCGAAAAGTGCGAGGTGAATGGCGAGAAGGCGCATCCGCTCTTCGCCTT         480
MH               CTTTATGCTCTTCGAAAAGTGCGAGGTGAATGGCGAGAAGGCGCATCCGCTCTTCGCCTT         480
TB               CTTTATGCTCTTCGAAAAGTGCGAGGTGAATGGCGAGAAGGCGCATCCGCTCTTCGCCTT         480
MB               CTTTATGCTCTTCGAAAAGTGCGAGGTGAATGGCGAGAAGGCGCATCCGCTCTTCGCCTT         480
                 ************************************************************

NM_174076.3      CCTTCGGGAGGTTCTGCCCACGCCAAGTGACGACGCCACTGCTCTCATGACCGA      534
TH               CCTTCGGGAGGTTCTGCCCACGCCAAGTGACGACGCCACTGCTCTCATGACCGA      534
MH               CCTTCGGGAGGTTCTGCCCACGCCAAGTGACGACGCCACTGCTCTCATGACCGA      534
TB               CCTTCGGGAGGTTCTGCCCACGCCAAGTGACGACGCCACTGCTCTCATGACCGA      534
MB               CCTTCGGGAGGTTCTGCCCACGCCAAGTGACGACGCCACTGCTCTCATGACCGA      534
                 ******************************************************
```

Fig. S7. Representative DNA sequence alignment of *GPX1* gene (534-bp) among tolerant and mastitis affected Holstein and Brown Swiss dairy cows and reference sequence available in the GenBank gb|NM_174076.3|. TH= Tolerant Holstein; MH= Mastitic Holstein; TB= Tolerant Brown Swiss and MB= Mastitic Brown Swiss. The asterisks represent the similarity.

```
XM_005210409.4      TAAGAATTGTTTAAACTGAAAAAATAAGAAATTTTGTAATTTAAATGTACACTATGATTT      60
TH                  TAAGAATTGTTTAAACTGAAAAAATAAGAAATTTTGTAATTTAAATGTACACTATGATTT      60
MH                  TAAGAATTGTTTAAACTGAAAAAATAAGAAATTTTGTAATTTAAATGTACACTATGATTT      60
TB                  TAAGAATTGTTTAAACTGAAAAAATAAGAAATTTTGTAATTTAAATGTACACTATGATTT      60
MB                  TAAGAATTGTTTAAACTGAAAAAATAAGAAATTTTGTAATTTAAATGTACACTATGATTT      60
                    ************************************************************
XM_005210409.4      GGTTGTATACTCTCAAACAGCTTCTACTTCCCACCCCCAAGTTGCTTTTATTTAGTGAAC      120
TH                  GGTTGTATACTCTCAAACAGCTTCTACTTCCCACCCCCAAGTTGCTTTTATTTAGTGAAC      120
MH                  GGTTGTATACTCTCAAACAGCTTCTACTTCCCACCCCCAAGTTGCTTTTATTTAGTGAAC      120
TB                  GGTTGTATACTCTCAAACAGCTTCTACTTCCCACCCCCAAGTTGCTTTTATTTAGTGAAC      120
MB                  GGTTGTATACTCTCAAACAGCTTCTACTTCCCACCCCCAAGTTGCTTTTATTTAGTGAAC      120
                    ************************************************************
XM_005210409.4      AATTGTCGTCTTTTATTTTTCTTGAGTCTTAATATCTTTAGTGGGAAGTGTCCTAGGAAT      180
TH                  AATTGTCGTCTTTTATTTTTCTTGAGTCTTAATATCTTTAGTGGGAAGTGTCCTAGGAAT      180
MH                  AATTGTCGTCTTTTATTTTTCTTGAGTCTTAATATCTTTAGTGGGAAGTGTCCTAGGAAT      180
TB                  AATTGTCGTCTTTTATTTTTCTTGAGTCTTAATATCTTTAGTGGGAAGTGTCCTAGGAAT      180
MB                  AATTGTCGTCTTTTATTTTTCTTGAGTCTTAATATCTTTAGTGGGAAGTGTCCTAGGAAT      180
                    ************************************************************
XM_005210409.4      TCTGATGAAAAGTACAGCAAGATTAAAATATTGATAATTATTAGCAAGTATAATACTGCT      240
TH                  TCTGATGAAAAGTACAGCAAGATTAAAATATTGATAATTATTAGCAAGTATAATACTGCT      240
MH                  TCTGATGAAAAGTACAGCAAGATTAAAATATTGATAATTATTAGCAAGTATAATACTGCT      240
TB                  TCTGATGAAAAGTACAGCAAGATTAAAATATTGATAATTATTAGCAAGTATAATACTGCT      240
MB                  TCTGATGAAAAGTACAGCAAGATTAAAATATTGATAATTATTAGCAAGTATAATACTGCT      240
                    ************************************************************
XM_005210409.4      TTGAACTTAAAATACGTTTATAACAAGCATATAAAGGTCATACACAAAATATACTTTAAA      300
TH                  TTGAACTTAAAATACGTTTATAACAAGCATATAAAGGTCATACACAAAATATACTTTAAA      300
MH                  TTGAACTTAAAATACTTTTATAACAAGCATATAAAGGTCATACACAAAATATACTTTAAA      300
TB                  TTGAACTTAAAATACTTTTATAACAAGCATATAAAGGTCATACACAAAATATACTTTGAA      300
MB                  TTGAACTTAAAATACGTTTATAACAAGCATATAAAGGTCATACACAAAATATACTTTAAA      300
                    *************** ***************************************** **
XM_005210409.4      GTGTCCAAAAAATCTGAATCTATTTTCATGTGTATACAGACAGAAATCATGTTTTTAATAA      360
TH                  GTGTCCAAAAAATCTGAATCTATTTTCATGTGTATACAGACAGAAATCATGTTTTTAATAA      360
MH                  GTGTCCAAAAAATCTGAATCTATTTTCATGTGTATACAGACAGAAATCATGTTTTTAATAA      360
TB                  GTGTCCAAAAAATCTGAATCTATTTTCATGTGTATACAGACAGAAATCATGTTTTTAATAA      360
MB                  GTGTCCAAAAAATCTGAATCTATTTTCATGTGTATACAGACAGAAATCATGTTTTTAATAA      360
                    ************************************************************
XM_005210409.4      TGGAAATGGGATAGCTCATAAAAAATTAAAATTAAGGTGTTTTGGACTATTTTATTAAGTA      420
TH                  TGGAAATGGGATAGCTCATAAAAAATTAAAATTAAGGTGTTTTGGACTATTTTATTAAGTA      420
MH                  TGGAAATGGGATAGCTCATAAAAAATTAAAATTAAGGTGTTTTGGACTATTTTATTAAGTA      420
TB                  TGGAAATGGGATAGCTCATAAAAAATTAAAATTAAGGTGTTTTGGACTATTTTATTAAGTA      420
MB                  TGGAAATGGGATAGCTCATAAAAAATTAAAATTAAGGTGTTTTGGACTATTTTATTAAGTA      420
                    ************************************************************
XM_005210409.4      AGGAAACATTGGTGGCCCACATCCCTTTATTAACTTTAGACTTAAAACTGCTGAATCATA      480
TH                  AGGAAACATTGGTGGCCCACATCCCTTTATTAACTTTAGACTTAAAACTGCTGAATCATA      480
MH                  AGGAAACATTGGTGGCCCACATCCCTTTATTAACTTTAGACTTAAAACTGCTGAATCATA      480
TB                  AGGAAACATTGGTGGCCCACATCCCTTTATTAACTTTAGACTTAAAACTGCTGAATCATA      480
MB                  AGGAAACATTGGTGGCCCACATCCCTTTATTAACTTTAGACTTAAAACTGCTGAATCATA      480
                    ************************************************************
```

Fig. S8. Representative DNA sequence alignment of *AhpC/TSA* gene (480-bp) among tolerant and mastitis affected Holstein and Brown Swiss dairy cows and reference sequence available in the GenBank gb|XM_005210409.4|. TH= Tolerant Holstein; MH= Mastitic Holstein; TB= Tolerant Brown Swiss and MB= Mastitic Brown Swiss. The asterisks represent the similarity.
